# Robotic-based ACTive somatoSENSory (Act.Sens) retraining on upper limb functions with chronic stroke survivors: study protocol for a pilot randomised controlled trial

**DOI:** 10.1186/s40814-021-00948-3

**Published:** 2021-11-15

**Authors:** Ananda Sidarta, Yu Chin Lim, Christopher Wee Keong Kuah, Yong Joo Loh, Wei Tech Ang

**Affiliations:** 1 Rehabilitation Research Institute of Singapore, Nanyang Technological University, Singapore, Singapore; 2 Centre for Advanced Rehabilitation Therapeutics (CART), Tan Tock Seng Hospital, Singapore, Singapore; 3 Department of Rehabilitation Medicine, Tan Tock Seng Hospital, Singapore, Singapore; 4 School of Mechanical & Aerospace Engineering, Nanyang Technological University, Singapore, Singapore; 5 Lee Kong Chian School of Medicine, Nanyang Technological University, Singapore, Singapore

**Keywords:** Proprioception, Kinaesthesia, Robot-assisted training, Haptic guidance, Reward feedback

## Abstract

**Background:**

Prior studies have established that senses of the limb position in space (proprioception and kinaesthesia) are important for motor control and learning. Although nearly one-half of stroke patients have impairment in the ability to sense their movements, somatosensory retraining focusing on proprioception and kinaesthesia is often overlooked. Interventions that simultaneously target motor and somatosensory components are thought to be useful for relearning somatosensory functions while increasing mobility of the affected limb. For over a decade, robotic technology has been incorporated in stroke rehabilitation for more controlled therapy intensity, duration, and frequency. This pilot randomised controlled trial introduces a compact robotic-based upper-limb reaching task that retrains proprioception and kinaesthesia concurrently.

**Methods:**

Thirty first-ever chronic stroke survivors (> 6-month post-stroke) will be randomly assigned to either a treatment or a control group. Over a 5-week period, the treatment group will receive 15 training sessions for about an hour per session. Robot-generated haptic guidance will be provided along the movement path as somatosensory cues while moving. Audio-visual feedback will appear following every successful movement as a reward. For the same duration, the control group will complete similar robotic training but without the vision occluded and robot-generated cues. Baseline, post-day 1, and post-day 30 assessments will be performed, where the last two sessions will be conducted after the last training session. Robotic-based performance indices and clinical assessments of upper limb functions after stroke will be used to acquire primary and secondary outcome measures respectively. This work will provide insights into the feasibility of such robot-assisted training clinically.

**Discussion:**

The current work presents a study protocol to retrain upper-limb somatosensory and motor functions using robot-based rehabilitation for community-dwelling stroke survivors. The training promotes active use of the affected arm while at the same time enhances somatosensory input through augmented feedback. The outcomes of this study will provide preliminary data and help inform the clinicians on the feasibility and practicality of the proposed exercise.

**Trial registration:**

ClinicalTrials.gov NCT04490655. Registered 29 July 2020.

**Supplementary Information:**

The online version contains supplementary material available at 10.1186/s40814-021-00948-3.

## Introduction

Stroke is among the top three leading causes of long-term disability worldwide [1]. The resulting impairment of motor functions, particularly in the upper limb (UL), may lead to dependency in activities of daily living (ADL) [2, 3]. In addition to motor impairments, approximately 50% of stroke survivors also suffer from somatosensory impairments, which have a significant and long-lasting impact on their motor recovery and quality of life [4–7]. For example, Carey, Matyas and Baum [7] revealed that stroke survivors with somatosensory loss showed a significant decrease in retained participation across the domains of instrumental, social and physical leisure activities, compared to those without somatosensory loss.

Despite the scarce evidence, training paradigms that simultaneously combine both motor and somatosensory components are thought to be more beneficial for stroke survivors [8–10] owing to the significant correlation between the two impairments following stroke [11]. Hence, these forms of training paradigm are useful for relearning somatosensory functions while increasing the mobility of the affected UL, since stroke survivors with UL impairments are less likely to use their hands, which subsequently leads to reduced sensory processing in their UL [12].

Proprioception and kinaesthesia are the senses that provide sensory information of limb position in space. Studies in healthy adults have demonstrated the importance of passive training of proprioception in motor learning, where such training is useful for enhancing and facilitating motor performance and learning [13–16]. In stroke survivors, there is evidence highlighting the role of proprioception as a predictor for motor relearning [17, 18]. For instance, the extent of motor relearning is associated with proprioceptive impairment, in which damage to the somatosensory areas in the brain could impair learning ability and thus interfere with the recovery of sensory-guided movements [19]. A randomised controlled trial (RCT) with a variety of tactile and proprioceptive training has shown that such forms of intervention are helpful for the recovery of sensation after stroke [20]. Given the importance of proprioception in motor control and learning, the retraining of tactile sensation alone after stroke may not be sufficient to improve UL motor functions [21]. Accordingly, the inclusion of somatosensory-based training focusing on proprioception and kinaesthesia deserves more attention, while its role as an integral part of a stroke rehabilitation programme is often overshadowed by motor-based therapy.

To date, the use of robotic technology has shown great promise in the domain of stroke rehabilitation research [22, 23]. Robotic technology has also gained popularity for assessing UL somatosensory functions (proprioception and kinaesthesia) due to its objective quantification of performance and high interrater reliability [24–27]. Indeed, the use of robotic devices in stroke rehabilitation is clinically attractive as it can achieve more controlled therapy intensity, duration, and frequency. Specifically, robotic-based training of proprioception in chronic stroke survivors has been examined in [28]. In fact, a single session of passive somatosensory discrimination task is able to induce plasticity in the sensorimotor networks of the brain that correlate with the initial impairment [29].

The purpose of the current work is twofold. First, we assess whether the robotic-based intervention that integrates somatosensory components into the motor task can reliably bring benefits to stroke survivors compared to motor training alone. While some earlier studies emphasised the tactile or haptic aspects of distal joints [20, 21], this work focuses on the proprioception, kinaesthesia, and movement-induced cutaneous sensation of proximal joints (elbow and shoulder). The proposed task promotes active use of the affected UL of the participants, while at the same time enhances somatosensory information through haptic guidance, unlike some prior studies which use a purely passive discrimination task (e.g. in [29]). Second, the results of this study will be valuable to estimate the effect size and to inform a decision for larger-scale multi-centre studies in the future, since the existing evidence of similar training paradigms is still insufficient [9]. Taken together, this work will add to the knowledge of the feasibility and benefits of a combined robotic-based somatosensory and motor retraining post-stroke.

## Methods/design

### Study design

**Fig. 1:**
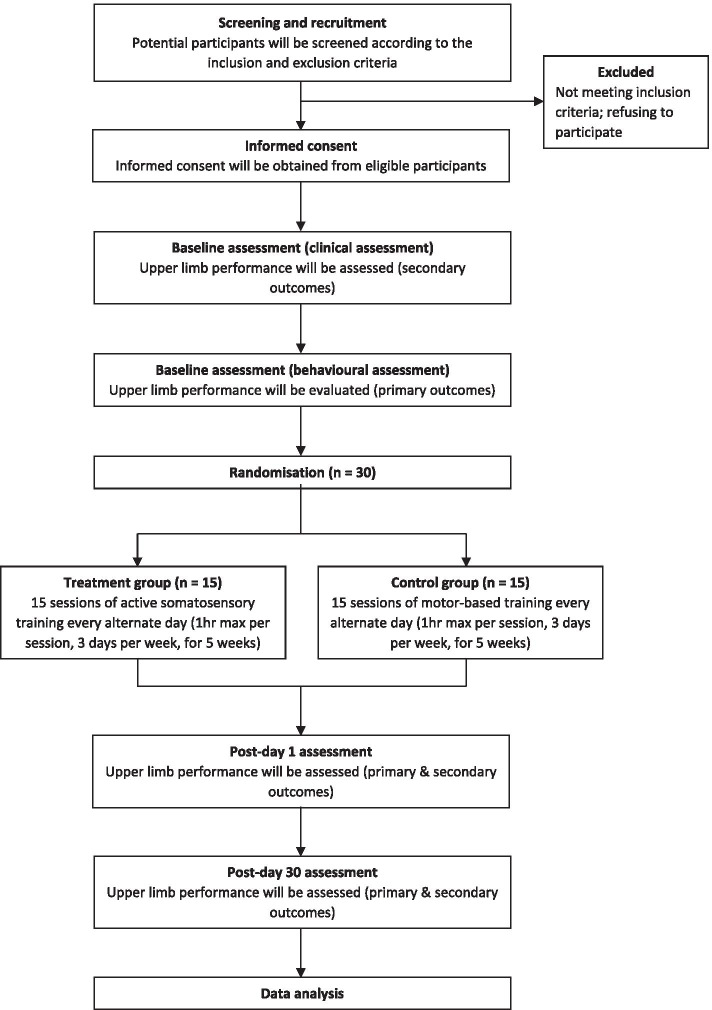
Diagram of study flow

**Fig. 2:**
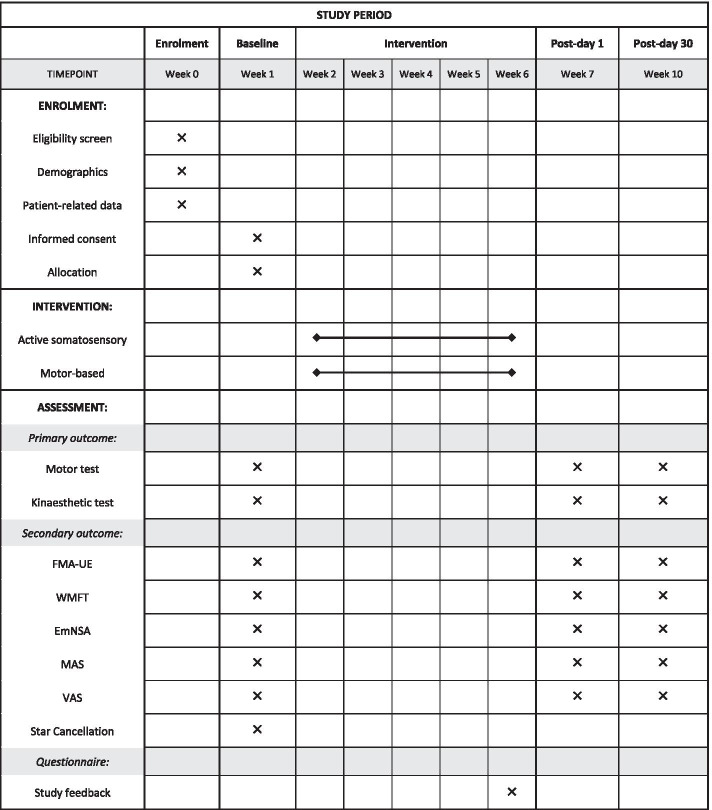
SPIRIT diagram of the schedule of enrolment, interventions, and outcome measures. Abbreviations: *FMA-UE* Fugl-Meyer Assessment for Upper Extremity, *WMFT* streamlined Wolf Motor Function Test, *EmNSA* Erasmus MC modifications to the Nottingham Sensory Assessment, *MAS* modified Ashworth scale of spasticity

An intensive robotic-based behavioural training for chronic stroke survivors, with emphases on retraining of proprioception and kinaesthesia, will be conducted. This is a two-arm RCT consisting of treatment and control groups. Participants are chronic stroke survivors from the local community in Singapore who will go through a series of training using their affected UL for 15 regular sessions. Each session will take place every alternate day, 3 days per week, for 5 weeks. On top of the training sessions, three behavioural and clinical assessments will be performed at baseline, post-day 1, and post-day 30, where the follow-up assessments will happen the following day and 30 days after the last training session respectively. All assessments will be administered by a therapist who is blinded to treatment allocation throughout the study. This therapist is different from the person who will be responsible for the routine intervention. Prior to the commencement of the study, the principal investigator will provide a half-day training session to the therapists involved. The process flow of this study is outlined in Fig 1, and the timeline of recruitment and enrolment processes, interventions, and study visits is summarised in the Standard Protocol Items: Recommendations for Interventional Trials (SPIRIT) diagram (Fig 2). This study was designed following the guidelines of the SPIRIT 2013 checklist (see Additional file 1).

### Participants

First-ever ischemic or haemorrhagic stroke survivors of at least 6-month post-stroke, between the ages of 21 and 75 years will be recruited from the local community rehabilitation centres. Stroke survivors with sensory impairment as assessed by the Erasmus MC modifications to the Nottingham Sensory Assessment (EmNSA) (each category ≤ 6/8), shoulder abduction and elbow extension of motor power grade > 2 as rated by the Medical Research Council (MRC) scale for muscle strength will be considered eligible to participate. However, those with bilateral impairment, high upper-limb spasticity (modified Ashworth scale of spasticity > 2), unilateral neglect as assessed by Star Cancellation Test (score < 44), cognitive impairment as examined by Mini-Mental State Examination (< 26/30), known history of mental disorders, and the inability to perform upper limb activity due to excessive pain will be excluded. Eligible participants will be randomly assigned to either a treatment group or a control group by a study team member not involved in the training or assessment. Block randomisation technique will be used to generate an allocation list with equal allocation ratio. Following this, the allocation list will be revealed in sealed envelopes to the therapist before the first training session. Participants in both groups who have completed the full study will receive financial compensation ($40/session) at the end of the last assessment session.

Potential participants will be recruited via face-to-face contact by on-site therapists who are part of the study team, where study rationale, potential benefits and time commitment will be introduced. This intervention would replace the UL therapy session of the participants on that specific day. However, they will not be asked to stop any UL intervention on the days they are not attending our programme. They may withdraw at any time from their participation for any reason and without any negative consequences for their rehabilitation in the future. To ensure a low dropout rate, all participants will be given a list of their scheduled visit in advance and followed up regularly by the study team through phone calls as a reminder of their upcoming visit. The study team will also keep a record of participant attendance to track the retention rate, and other UL exercises that the participants have been doing.

### Sample size

To estimate the sample size for this study, a Cohen’s *d* effect size of 0.5 was applied with a statistical power of 0.80 and an alpha level of 0.05. The resulting estimate is 28 participants in total based on the statistical power analysis programme G*Power (version 3.1.9.7). Approximately 36 participants will be screened and recruited, where 30 subjects will be estimated to complete the full study (with a conservative dropout rate of 20%).

### Equipment

**Fig. 3:**
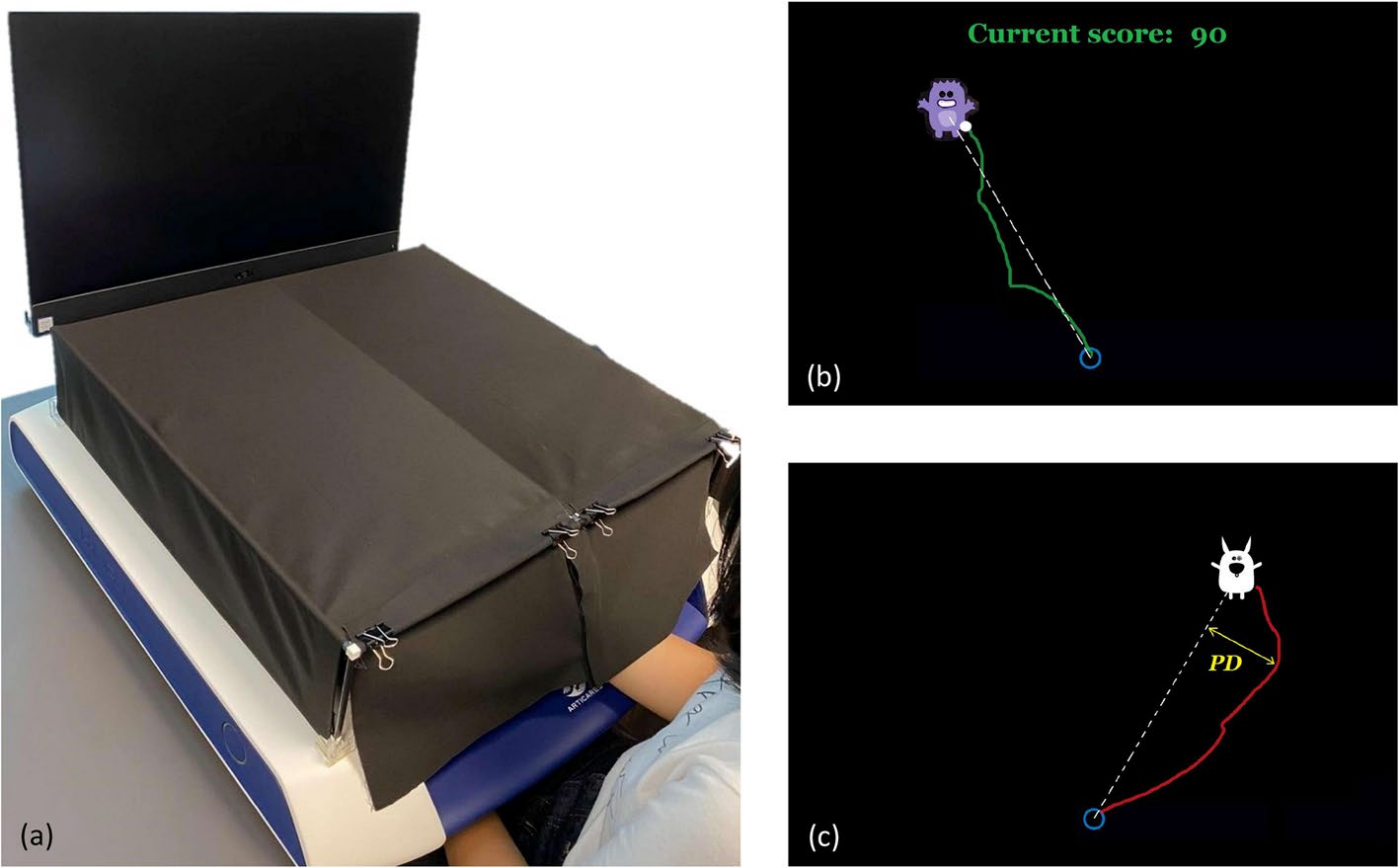
Experimental setup used in the study. **a** A compact table-top rehabilitation robotic device with the rectangular box covering the view of the affected arm. An example of feedback shown on the LCD display following a successful trial (**b**) and unsuccessful trial (**c**), respectively. Note: the person depicted is not a patient, but a study team member

This study will employ a planar or 2-dimensional (2D) table-top rehabilitation robotic device (H-Man, Articares Pte Ltd) [30] that has a robotic handle resembling an ergonomic computer mouse. The robotic device will be placed on a height-adjustable table and linked via a network cable to a computer with a 24-inch LCD display (Dell OptiPlex 7470 AIO, Intel Core i7-9700 with 3 GHz CPU, 16 GB RAM, and 64-bit Windows 10). The 2D coordinate of the handle will be captured and recorded by the computer through a custom-made software coded in MATLAB R2019b (MathWorks Inc., Natick, MA, USA). The software also provides an interactive gaming interface which corresponds to the 2D workspace of the robotic device, shown on the LCD display. An ergonomic upper limb support (MoMo, Reharo Corp.) will be used to provide support to the elbow to prevent fatigue. A custom-made rectangular box is placed on top of the robotic setup to block the vision of the arm (Fig. 3a). Participants will be seated on a clinical chair in front of the robot with their body securely strapped onto the chair. The affected elbow will be flexed to ∼90°, the forearm pronated to ~45°, and the hand gently placed on the robotic handle secured with a Velcro strap. The initial position of this handle, which is in the middle of the horizontal axis of the workspace, determines the start location during the session. The body alignment will be set in such a way that the handle is roughly in front of the affected shoulder midline.

### Intervention design

Participants in each group will receive 15 individual, face-to-face sessions of robotic-based training by a certified therapist for a maximum of one hour per session. The same therapist will be in charge of administering the whole intervention sessions to every participant and assuring adherence to the protocol. Each training session will begin with a warm-up exercise, after which participants will continue with the active somatosensory training task or motor-based control task depending on the group they are assigned to. In the first training session, there will be some familiarisation trials with instructions for ten repetitions before the actual training commences.

#### Warm-up exercise

This session entails two warm-up exercises with the use of robotic device to prepare the sensorimotor system for the subsequent training task. On the LCD display, a black screen will be shown together with a circular indicator to denote a start position of the movement (1.2 cm diameter, 2 mm thickness). A smaller white circle (0.8 cm diameter) will represent the position of the handle in the workspace, which will always be shown on the LCD display. To familiarise with the robotic setup, participants will begin the session by moving the robotic handle towards a visual object shown on the monitor screen, according to the range of motion of their UL for about 5 min. Following this, the robotic handle will move back to the start position again, after which participants will perform the second warm-up exercise that mimics the joint approximation technique. In this exercise, the robot will produce a spring-like resistive force (stiffness = 900 N/m) which is position-dependent while the participants are moving in the direction of the target, meaning the resistance will increase as the handle is getting nearer to the target location. They will be told to pay attention to the resistive force preventing them to move towards the corresponding target location. The distance between the two adjacent points is 10 cm. Audio feedback will be provided if the movement has reached the target, after which the position will be locked for 2 s before starting the next repetition. The duration of this task is 16 repetitions.

#### Active somatosensory training task

The treatment group will participate in the active somatosensory training as follows. Participants will be instructed to propel the robotic handle with their affected UL from the start position to one of the target positions. On the LCD display, a gaming interface will be shown together with the white handle position and the start position indicator. However, they will be informed that this white circle will disappear once the handle is moved 2 cm away from the centre of the start position. There will be four different target positions located 15 cm equidistant from the start position, at an angle of 30°, 60°, 120°, and 150° with respect to the horizontal axis. Each of these positions will be presented an equal number of times in one of the two fixed, repeated sequences that either starts from 30° to 150° or from 150° to 30°. The sequences will be alternatively varied across blocks. A circular visual target in the form of a cartoon character will appear at one of the positions as shown on the LCD display, with a 440 Hz tone as a movement initiation cue. Participants will begin reaching towards the target only after this cue and will be asked to produce the movement as straight as possible. Participants will be told about the augmented feedback that will help them learn the desired path to reach the target (see below, ‘Augmented feedback’). The view of the active forearm will be occluded using a rectangular box, and there is no visual indicator of the handle whatsoever on the LCD display. Thus, they will perform the task by relying more on somatosensory cues of their upper limb in space.

Once the movement is completed the robot will hold the position for 2 s, during which augmented feedback will be given in two kinds. Following the feedback, the robot will bring the hand back to the start position in 10 cm/s while the UL remains relaxed. This returned trajectory will follow a smooth and straight path according to the minimum jerk model in [31] (stiffness = 3500 N/m, damping = 20 N.s/m). The next repetition will continue after a brief interval of 1.5 s. Each session comprises ten blocks of 24 repetitions. Note that participants will be given a short break in between consecutive blocks, or if the therapist notices decreased movement quality in them.

#### Augmented feedback

Prior studies which enhance somatosensory information and provide positive feedback have uncovered less movement error and improved somatosensory acuity in participants by reinforcing successful performance and providing motivational boost [15, 16]. In a similar fashion, the current training will be facilitated by the presence of two kinds of augmented feedback (Table 1). The first feedback will be available concurrently while the participants perform the movement. This online feedback will be perceived as a ‘virtual wall’ produced by the robot handle (stiffness = 1000 N/m) along the path connecting the start position and the target. Such kind of haptic feedback can serve as somatosensory cues to the active arm while participants are moving towards the corresponding target. If the movement deviates too much from the trajectory, they will feel a cushion-like force preventing the handle to move further away from the ideal path.

**Table 1:** Augmented feedback used during training

Type of feedback	Content (source)	Schedule	Purpose	Treatment	Control
Somatosensory cues	Virtual wall during movement (force produced by the robot handle)	Concurrent	Haptic guidance, through feeling to enhance participation	√	
Visual cues	Handle position as a cursor (on LCD display) and vision of the affected arm	Concurrent	Provide hand position in real-time		√
Performance	Trajectory of the previous movement (on LCD display)	Terminal (successful and unsuccessful)	Improve subsequent performance, and for • corrective action	√	√
Outcome	Pleasant tone, scores, words of encouragement (LCD display and speaker)	Terminal (successful)	• Motivation, positive reinforcement	√	√

The second feedback will be available at the end of each movement that informs the participants how good the latest movement has been performed. This terminal feedback will be displayed as a trajectory of the movement made, together with a reference line connecting the start position and the target centre. Another kind of terminal feedback will be given only following every successful movement outcome as positive feedback. Here, a green-coloured text with a running score will be shown on the LCD display together with a pleasant audio tone. At random intervals, positive words in the form of audio feedback (e.g. ‘Good job!’, ‘Well done!’, or ‘Excellent!’) will also be played as additional reward or reinforcement. No positive feedback will be given if the movement is unsuccessful, although the handle trajectory will still be shown as information to improve subsequent performance.

The size of the visual targets for reaching will determine the provision of positive feedback after each successful outcome. A movement outcome is considered successful based on two criteria, i.e. if the endpoint error *and* lateral perpendicular deviation are within the span of the target. The diameter of each target is set to 3.0 cm and will remain fixed across sessions.

#### Motor-based control task

A control group will be introduced as a comparison to examine whether any observed improvements in the performance are strictly due to the proposed training task. Participants in this group will also take part in the robotic-based training with some important differences. The same centre-out reaching movements will be used, but without any emphasis on proprioception or kinaesthesia. Here, the view of the forearm will not be blocked, and the handle position will always be shown on the LCD display (Table 1). Other feedback such as running score and positive feedback will still be provided to inform the participants of their trial outcomes, but no somatosensory cues (haptic guidance) will be given while they are actively moving. They will complete this motor-based training for the same number of sessions and repetitions as the treatment group, with the same assessments conducted before and after the whole training.

### Primary outcome measures

Two robotic-based tests will generate behavioural performance indices as the primary outcome measures of participants’ affected UL before (baseline) and after training (post-day 1 and post-day 30).

#### Motor performance test

The motor test will evaluate the movement quality of the affected UL during planar reaching. This test will be designed and conducted in similar fashion as the training, yet no augmented feedback whatsoever will be delivered to the participants regarding their performance. The visual target of this test will also be presented one at a time at random and correspond to the targets’ positions in the training section described earlier. All visual targets in the assessment will have a diameter of 1.5 cm. The vision of their affected forearm will not be blocked. This test comprises 20 repetitions in total.

#### Kinaesthetic test

Following the motor test, participants will perform the kinaesthetic test that is essentially a joint position matching task [32], which requires them to reproduce a reference movement presented to the affected arm. The test evaluates the ability to dynamically feel the sensation of movement, as well as, to perceive the endpoint position of the reference trajectory in the absence of the vision of their affected forearm.

In the beginning of every trial, participants will be instructed to remain relaxed while the robot displaces their hand forward and backward towards a certain target position with a smooth and straight trajectory production, with a speed of 10 cm/s. Once the robot completes this reference movement, the participants will be asked to reproduce the previous movement they just experienced, including to match the same speed as much as they can. Participants will only begin their movement after getting the movement initiation cues appearing for 1.2 s. This assessment will last for 20 repetitions. In the second block, a passive version of the matching task will be administered where the ability to sense the endpoint position of the movement will be examined. Here, after the robot presents the reference movement the participants will remain relaxed as the robot will displace again the arm in the same direction with a speed of 2 cm/s [18]. They will be required to indicate the endpoint of the reference movement by pressing the ‘Enter’ key on a keyboard. This task again consists of 20 repetitions. As with the motor test, participants will not receive any augmented feedback (haptic or reward) throughout this test.

### Secondary outcome measures

Several clinical assessments of UL function post-stroke will be exploited to evaluate participants’ UL performance before (baseline) and after training (post-day 1 and post-day 30). The motor section of Fugl-Meyer Assessment for Upper Extremity (FMA-UE) will be used to measure movement ability across the domains of reflex, movement, and coordination [33]. Participants’ functional ability will also be assessed using the streamlined Wolf Motor Function Test (WMFT) which includes six timed tasks: hand to table (front), hand to box (front), lift can, lift pencil, fold towel, and reach and retrieve [34]. The EmNSA will be employed to evaluate UL sensation (exteroception) through a wide range of subscales that include light touch, tactile discrimination, and proprioception [35]. Herein the effect of multiple training sessions on other somatosensory modalities such as tactile sensation and sharp-blunt discrimination will be examined. Additionally, both modified Ashworth scale (MAS) of spasticity [36] and visual analogue scale (VAS) [37] will be performed to monitor upper limb spasticity and pain intensity in participants, respectively. Note that the Star Cancellation Test [38] will merely be conducted at baseline to identify the presence of unilateral neglect in participants.

### Short study questionnaire

A short, hardcopy questionnaire that consists of seven Likert scale questions will be administered at the end of the last training. The purpose of this questionnaire is to obtain individual opinions on the robotic-based training from the participants. This questionnaire will be completed by both groups of participants based on their experience of participating in the proposed training. In the questionnaire, participants will be asked to express their agreement or disagreement with various statements, e.g. ‘I feel the long-term benefits of this therapy’ and ‘I feel satisfied when I got reward feedback during the session’, using a 7-point scale ranging from strongly disagree to strongly agree.

### Statistical analysis

All statistical analyses will be carried out using SPSS (ver. 28.0). Information obtained from the primary and secondary assessments will be recorded as text files. From the motor test data, different kinematic parameters will be computed: endpoint error and smoothness. Likewise, kinaesthetic performance will be estimated by the difference between the reference trajectory produced by the robot and the reproduced trajectory by the participants. Behavioural outcomes obtained from every training session will be computed to identify the improvements in kinematic parameters session by session: total number of successful outcomes and average endpoint error in reaching trajectories. Due to its superiority in analysing repeated-measures dataset with and without missing values, a mixed-effects model with random intercepts and slopes will be used to reveal significant differences in the outcome measures between the two groups over three time points (baseline, post day 1, and post day 30). Statistical significance will be based on *p* value threshold of 0.05. Any post hoc comparisons will be performed following the main analysis using Tukey’s tests. Cohen’s *d* will be employed to estimate the effect size of the change scores for this study. Questionnaire results will be reported via descriptive statistics (frequency analysis).

All results will be presented with 95% confidence intervals.

### Data management

The study has been approved by the Institutional Review Board (IRB) of the Nanyang Technological University, Singapore (IRB-2019-10-022). As part of the screening process, basic demographic information (e.g., age, gender, and ethnicity) and health information (e.g., handedness, side and type of stroke) will be recorded. Data collected will be de-identified using unique study code numbers. To maintain the privacy of the participants, any report of individual data will only consist of performance measures without any name, address, or identifying information which complies with the university IRB guidelines. All patient-related information and data generated by the robotic system will be maintained on a secure server owned by the university. Data monitoring will comply with the university policy, guidelines, and data management plan (DMP) approved for the study. At the completion of the study, the results will be submitted for publication in a peer-reviewed journal and presented at national and international conferences.

### Adverse events

Although the prescribed UL training is not expected to result in any serious harm, the study team member will prepare both adverse events logbook and participant logbook for every participant. Any unexpected adverse events will be recorded in the adverse events logbook and reported to the IRB Committee. A follow-up meeting involving the principal investigator, the therapist in-charge and relevant team members will be instigated to ensure proper management of any issues. For routine monitoring purposes, blood pressure readings prior to and after every session, patients’ feedback, and discomfort experienced during and after the training (such as stiff shoulder and elbow pain) will be documented in the logbook by the therapist and the study member present during the session.

## Discussions

We present a repetitive robotic-based training protocol that retrains proprioception and kinaesthesia while at the same time promotes active use of the affected UL of community-dwelling chronic stroke survivors. The study will evaluate if the proposed protocol will be feasible and practical in the clinical setting. It is expected on average that the training will yield quantifiable improvements in somatosensory along with motor functions. While some earlier studies emphasised the tactile or haptic aspects of distal joints [20, 21], this work focuses on the proprioception, kinaesthesia, and movement-induced cutaneous sensation of proximal joints (elbow and shoulder). The tasks presented here require active participation of the participants, unlike some prior studies which make use of a purely passive somatosensory discrimination task [29]. A recent systematic review evidence [9] suggested that exercises that synchronously combine motor and somatosensory retraining tend to elicit stronger connections between the motor and somatosensory cortices compared to a paradigm that combines motor and somatosensory retraining sequentially, leading to increased neuroplasticity in the sensorimotor regions. Hence, both somatosensory and motor components are incorporated in this study within the same training task.

On top of having a task paradigm that could specifically address the impairments, the training paradigm with the incorporation of training strategies which are helpful for enhancing motor learning and the use of affected UL appears to be more effective [39]. In stroke survivors, the presence of somatosensory deficits poses difficulties in position and movement sense, which can be partially compensated by vision. In our task, however, blocking the view of the arm or handle will increase reliance on residual somatosensation of the affected UL, which can be challenging for the task completion given the underlying sensory deficits. As a result, we introduce the virtual wall along the path connecting the start position and the target as haptic feedback to enhance somatosensory inputs while participants are actively moving. Notably, the feedback will assist the movement by pushing the handle back from a deviated location to the desired trajectory. In addition, audio-visual terminal feedback will be provided at the end of every movement to increase the rewarding effect in practising the affected UL and to improve their subsequent behavioural performance.

Initial stroke severity has significant impacts on the mortality rate and influence functional outcomes measured 3 months later [40]. Indeed, patients with higher corticospinal tract integrity at the onset were found to show better motor recovery in accordance with the proportional recovery rule [41, 42]. This study protocol recruits chronic stroke survivors whose medical conditions are more stable. It is likely that patients who have higher motor and somatosensory scores (i.e. mild impairments) may exhibit ceiling effects such that no further improvements can be attained. Therefore, participants recruited in our study are those with moderate to severe somatosensory impairments.

In brief, the positive outcomes of this RCT will be informative for future research and RCT. The important implications include emphasising the feasibility of a combined robot-assisted somatosensory and motor retraining of UL post-stroke, and providing insights into the effective implementation of such rehabilitation exercise in the community setting. Results from this study may also add to existing knowledge on whether employing somatosensory training components in rehabilitation could generate greater improvements in functional recovery than the motor-based training alone.

## Trial status

The recruitment of potential participants will commence in January 2021, and the study trial is expected to continue until 31 March 2022.

## Supplementary Information

**Additional file 1.** SPIRIT Checklist.

## Data Availability

Data collected and published will be de-identified to maintain the privacy of the participants, any report of individual data will only consist of performance measures. Upon completion of the study, the dataset will be available from the corresponding author upon reasonable request.

## Abbreviations

2D: Two-dimensional
ADL: Activities of daily living
Em-NSA: Erasmus MC modifications to the Nottingham Sensory Assessment
FMA-UE: Fugl-Meyer Assessment for Upper Extremity
LCD: Liquid crystal display (computer display)
MMSE: Mini-Mental State Examination
MRC: Medical Research Council
MSA: Modified Ashworth scale (of spasticity)
RCT: Randomised controlled trial
SPIRIT: Standard Protocol Items: Recommendations for Interventional Trials
UL: Upper limb
VAS: Visual analogue scale
WFMT: Streamlined Wolf Motor Function Test
SPIRIT: Standard Protocol Items: Recommendations for Interventional Trials
